# Cytoreductive Surgery plus Hyperthermic Intraperitoneal Chemotherapy Improves Survival with Acceptable Safety for Advanced Ovarian Cancer: A Clinical Study of 100 Patients

**DOI:** 10.1155/2021/5533134

**Published:** 2021-06-22

**Authors:** Jue Zhang, Xin-bao Li, Zhong-he Ji, Ru Ma, Wen-pei Bai, Yan Li

**Affiliations:** ^1^Department of Peritoneal Cancer Surgery, Beijing Shijitan Hospital, Capital Medical University, China; ^2^Department of Gynecology, Beijing Shijitan Hospital, Capital Medical University, China

## Abstract

**Background:**

The mainstay of treatment for advanced ovarian cancer is debulking surgery followed by chemotherapy that includes carboplatin and paclitaxel, but the prognosis is poor. This study is aimed at evaluating the efficacy and safety of cytoreductive surgery plus hyperthermic intraperitoneal chemotherapy (CRS+HIPEC) as first-line surgical treatment in patients with advanced ovarian cancer (AOC).

**Methods:**

FIGO stage III/IV AOC patients underwent CRS+HIPEC as first-line surgical treatment at our center from December 2007 to January 2020. The primary endpoint was survival, and the secondary endpoint was safety.

**Results:**

Among 100 patients, the median Karnofsky performance status (KPS) score was 80 (50-100), median peritoneal cancer index (PCI) was 19 (1-39), median completeness of cytoreduction (CC) score was 1 (0-3), number of organ regions removed was 4 (3-9), number of peritoneal regions removed was 4 (1-9), and number of anastomoses was 1 (0-4). The median follow-up was 36.8 months; 75 (75.0%) patients were still alive, and 25 (25.0%) had died. The median overall survival (mOS) was 87.6 (95% CI: 72.1-103.0) months, and the 1-, 2-, 3-, 4-, and 5-year survival rates were 94.1%, 77.2%, 68.2%, 64.2%, and 64.2%, respectively. Univariate analysis showed that better mOS correlated with an age ≤, KPS ≥ 80, ascites ≤ 1000 ml, PCI < 19, and CC score 0-1. Multivariate Cox analysis showed that CC was an independent factor for OS; patients who underwent CRS with a CC score 0-1 had a mPFS of 67.8 (95% CI: 48.3-87.4) months. The perioperative serious adverse event and morbidity rates were 4.0% and 2.0%, respectively.

**Conclusions:**

CRS+HIPEC improves survival for AOC patients with acceptable safety at experienced high-volume centers. Stringent patient selection and complete CRS are key factors for better survival.

## 1. Introduction

The majority of patients with ovarian cancer (OC) are diagnosed with advanced disease that has spread beyond the ovaries to cause peritoneal metastasis (PM), and this advanced stage accounts for the highest mortality of all gynecologic cancers [1]. The fatal manifestation of cancer dissemination on the omentum, peritoneum, and mesentery leads to refractory ascites, progressive intestinal obstruction, and intractable abdominal pain associated with early death and a miserable quality of life for such patients [2, 3]. Even after the standard treatment of optimal debulking surgery followed by intravenous platinum/taxane-based chemotherapy for advanced ovarian cancer (AOC) [4, 5], 75% of patients still develop recurrence and present with PM [6], which is the most difficult obstacle to improving AOC treatment.

Over the past three decades, aggressive cytoreductive surgery (CRS) plus hyperthermic intraperitoneal chemotherapy (HIPEC) has been developed as a comprehensive treatment package integrating multivisceral resections to remove macroscopic residual tumors, and HIPEC has been used to treat residual cancer cells after CRS [7, 8].

Bakrin et al. [9] report a multicenter retrospective cohort study included 566 patients, 92 patients with primary EOCPM, and 474 patients with recurrent EOCPM, which the CRS+HIPEC as first-line treatment. The median overall survivals (mOSs) were 35.4 months and 45.7 months for advanced and recurrent EOC, respectively. van Driel et al. [10] report a first RCT to compare the efficacy and safety of CRS+HIPEC and CRS groups, and the results showed the survival gain in the CRS+HIPEC group. Although CRS+HIPEC is considered one of several acceptable options for patients with stage III/IV OC by the National Comprehensive Cancer Network (NCCN) guidelines [11], there is currently limited evidence from clinical studies to definitively determine the efficacy and safety of standard operation programs and patient selection criteria.

A retrospective analysis was conducted in this study to assess the efficacy and safety of CRS+HIPEC as a front-line surgical regimen in 100 patients with AOC.

## 2. Methods and Materials

### 2.1. Patient Selection

From December 1, 2007, to January 1, 2020, 100 patients diagnosed with FIGO stage III/IV OC underwent CRS+HIPEC as a first-line surgery strategy at our center. Informed consent was obtained from all patients, and the study was approved by the institutional review board and the ethics committee.

The main inclusion criteria were as follows: (1) no history of PM-related surgery; (2) peripheral white blood cell count ≥ 3, 500/mm^3^ and platelet count ≥ 8, 000/mm^3^; (3) acceptable liver function, with bilirubin ≤ 2 × the upper limit of normal (ULN) and aspartic aminotransferase (AST) and alanine aminotransferase (ALT) ≤ 2 × ULN; (4) acceptable renal function, with serum creatinine (Scr) ≤ 1.5 mg/dl; (5) adequate cardiovascular and pulmonary function and function of other major organs that could tolerate major operation; and (6) Karnofsky performance status (KPS) ≥ 50.

The major exclusion criteria were as follows: (1) history of PM-related surgery; (2) any lung, liver, or prominent retroperitoneal lymph node metastases found during preoperative assessment; (3) imaging examination showing obvious contractures of the mesentery; (4) serum bilirubin level > 3 × ULN and ALT/AST ≥ 2 × ULN; (5) liver enzymes > 3 × ULN; (6) Scr > 1.5 mg/dl; (7) KPS score < 50; and (8) absence of peritoneal metastasis.

### 2.2. Preoperative Evaluation

Patients were evaluated according to the Chinese expert consensus [12] of CRS+HIPEC, which included the following: (1) physical examination: serum tumor marker levels including carbohydrate antigen 125 (CA 125, normal range: 0-35 U/mL), carbohydrate antigen 199 (CA 19-9, normal range: 0-37 U/mL), and carcinoembryonic antigen (CEA, normal range: 0-5 ng/mL); (2) imaging examination: abdominopelvic multidetector computed tomography (CT) plus multiplanar reconstruction to evaluate gastrointestinal motility, intestinal obstruction, and mesenteric contracture; and (3) cytology: cytological examination of ascites or exfoliating cells from peritoneal washing fluid.

### 2.3. CRS+HIPEC Procedures

All CRS+HIPEC procedures were conducted by a designated team focusing on PM treatment. Briefly, abdominal exploration was performed through a midline xiphoid-to-pubis incision after administering general anesthesia, and the peritoneal cancer index (PCI) was evaluated and recorded. Then, maximal CRS was performed, including curative or palliative resection of the primary tumor with acceptable margins an any involved adjacent structures, lymphadenectomy, and peritoneal resection, and then, the completeness of cytoreduction (CC) score was calculated.

Open HIPEC was implemented with each drug dissolved in 3 L of heated saline at 43 ± 0.5°C, and the duration of HIPEC was 60 min with a flow rate of 400 mL/min. The HIPEC regimens included docetaxel (DTX) 120 mg + cisplatin (DDP) 120 mg, DTX 120 mg + mitomycin C (MMC) 30 mg for patients with high-risk factors for renal dysfunction, and DTX 120 mg only for patients with a single kidney and/or impaired renal function confirmed by laboratory tests.

After the operation, the patients were transferred to the intensive care unit for recovery and then to the ward when they stabilized.

### 2.4. Postoperative Chemotherapy

Adjuvant chemotherapy was delivered within 6 to 8 weeks after CRS+HIPEC, including platinum/taxane-based systematic chemotherapy (SC) and perioperative intraperitoneal chemotherapy (IPC) through the IPC port once every 4 to 6 weeks. DDP 100 mg/m^2^ and paclitaxel/DTX 100 mg/m^2^ were administered.

### 2.5. Follow-Up

All patients were regularly followed up once every 3 months for the first 2 years, every 6 months for years 3 to 5, and every year thereafter to obtain detailed information on disease status. The most recent follow-up was performed on January 1, 2020, and no patients have been lost.

### 2.6. Study Parameters

(1) Clinicopathological characteristics: age, history of adjuvant therapy, KPS score, and preoperative tumor markers; (2) CRS+HIPEC-related parameters: duration of surgery, number of organs and peritoneal resected, number of anastomotic stoma, HIPEC regimens, PCI, CC score, and intraoperative volume; (3) survival: survival status, median overall survival (mOS), and median progression-free disease (mPFS); and (4) adverse events

### 2.7. Study Endpoints and Definition

**Figure 1:**
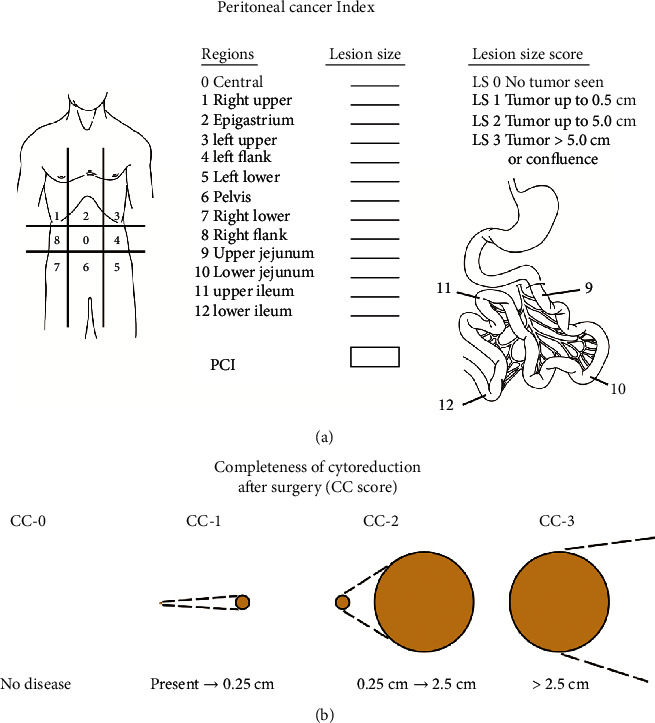
Graphical representation of PCI and CC score according to the Sugarbaker [8].

(1) The primary endpoints of this study were OS and PFS. OS was defined as the time interval from the first surgery to tumor-related death or last follow-up. PFS was calculated from the date of surgery until the last follow-up that met the following criteria: the patients who underwent surgery-based curative comprehensive treatment developed any clinical manifestations, the CA 125 level rose again after surgery, medical imaging discovered any mass in the operation field, and the biopsy confirmed the diagnosis. (2) The secondary endpoints were perioperative serious adverse events (SAEs), which were defined as complications directly attributable to the treatment within 30 days of CRS+HIPEC and were evaluated based on the National Cancer Institute Common Terminology Criteria for Adverse Events version 4.0 [11]. (3) The PCI, according to Sugarbaker's criteria [8], is a standardized intraoperative staging system to determine the PM burden. The abdomen was divided into 13 areas, which included 9 sections of the abdominal cavity and 4 sections of the upper ileum, lower ileum, upper jejunum, and lower jejunum. The size of intraperitoneal nodules in each area was quantified. A score of 0 indicates that no malignant deposits are visualized; a score of 1 signifies that tumor nodules ≤ 0.5 cm are present; a score of 2 indicates that tumor nodules between 0.5 and 5.0 cm are present; a score of 3 signifies that tumor nodules > 5.0 cm in any dimension are present, and a confluence or layering of the tumor is scored as 3. The maximum score is 39. (4) The CC score was defined as follows: a CC score of 0 indicates no visible residual peritoneal disease after CRS, a CC score of 1 indicates less than 2.5 mm of residual disease, a CC score of 2 indicates a residual tumor between 2.5 mm and 2.5 cm, and a CC score of 3 indicates more than 2.5 cm of residual tumor or the presence of a sheet of unresectable tumor nodules [8] (Figure 1)

## 3. Statistics Analysis

The patient information was systematically integrated into a prospectively established database. Data analysis was conducted using the Statistical Package for Social Sciences version 24.0 (SPSS, Inc., Chicago, IL). Descriptive data are expressed as medians [range or 95% confidence intervals (CIs)] for quantitative variables and as numbers (percentage) for qualitative data. The hypothesis test was performed by the *χ*^2^ test or Fisher's exact test. The Kaplan-Meier method was used to compare median survival with the log-rank test, and multivariate Cox regression analysis was performed to determine the independent predictors. The factors with *P* < 0.05 in the univariate analysis were included in the multivariate analysis model. A two-sided *P* < 0.05 was considered statistically significant.

## 4. Results

### 4.1. Characteristics of the AOC Patients

In total, 100 AOC patients were treated with 106 CRS+HIPEC procedures, including 6 patients who each underwent 2 CRS+HIPEC procedures due to tumor recurrence. The median age was 58.5 (28-87) years, and the median KPS score was 80 (50-100); according to histopathological classification, 91 (91/100, 91.0%) patients had serous adenocarcinoma, and 9 (9/100, 9.0%) patients had other types of tumors. For the areas of surgical excision, the median number of organ resections was 4 (3-9), and the number of peritoneal resections was 4 (1-9). Regarding adjuvant therapies, preoperatively, SC was applied in 48 (48/100, 48.0%) cases, and IPC was applied in 16 (16/100, 16.0%) cases; postoperatively, SC was applied in 76 (76/100, 76.0%) cases, and IPC was applied in 46.0 (46/100, 46.0%) cases. The clinicopathological characteristics are listed in Table 1.

### 4.2. Efficacy

**Figure 2:**
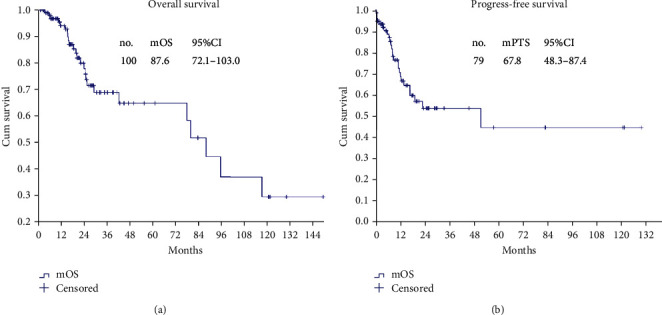
OS and PFS in the AOC patients. (a) OS of 100 AOC patients. (b) PFS of 79 AOC patients with complete CRS+HIPEC.

The median follow-up was 36.8 (0.8-159.3) months. At the time of analysis, 25 (25/100, 25%) patients had died, and 75 (75/100, 75.0%) patients were alive; the mOS was 87.6 (95% CI: 72.1-103.0) months, and the 1-, 2-, 3-, 4-, and 5- year survival rates were 94.1%, 77.2%, 68.2%, 64.2%, and 64.2%, respectively (Figure 2).

### 4.3. Univariate and Multivariate Analyses for Predictors of OS

**Figure 3:**
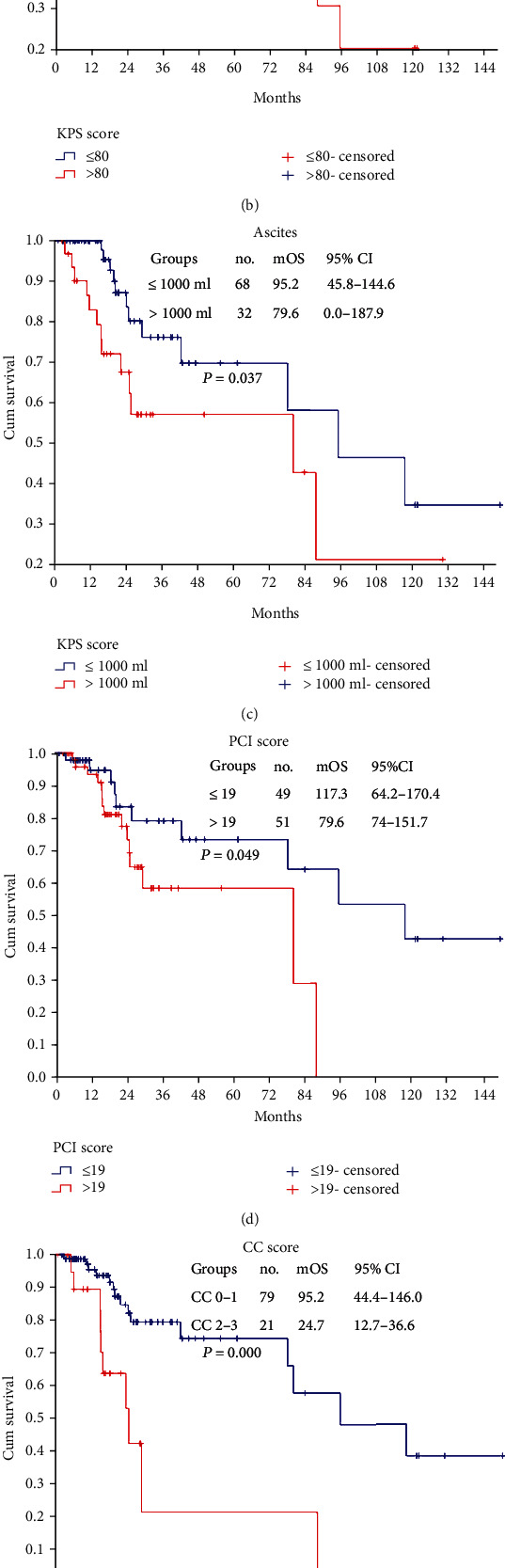
Overall survival of 100 AOC patients with correlation factors. (a) Age; (b) KPS score; (c) ascites; (d) PCI score; (e) CC score; (f) adverse events.

A univariate analysis identified 5 covariates indicative of improved survival, including age ≤ 58 years (Figure 3(a), *P* = 0.021), KPS score ≥ 80 (Figure 3(b), *P* = 0.015), ascites ≤ 1000 ml (Figure 3(c), *P* = 0.037), PCI ≤ 19 (Figure 3(d), *P* = 0.049), and CC score 0-1 (Figure 3(e), *P* < 0.001) (Table 2). Multivariate Cox regression analysis identified the CC score as the only independent predictor for better survival. Compared with a CC score of 2-3, a CC score of 0-1 was approximately 3.2 times (*P* = 0.009, HR = 3.2, 95%, and CI: 1.3-7.5) more likely to indicate improved survival (Table 2).

### 4.4. Special Analysis of Four Patients with an OS of over 10 Years

At the time of analysis, there were 4 (4/100, 4.0%) patients with an OS of over 10 years and without any evidence of tumor recurrence; their OS durations were 149.2, 129.9, 121.5, and 120.9 months. The detailed clinical course of these four patients is listed in Table 3.

### 4.5. Adverse Events

Adverse events (AEs) from grades I to V occurred in 31 (31/100, 31.0%) patients, including anemia and hypoproteinemia in 5 (5/100, 5.0%) patients, urinary fistula in 4 (4/100, 4.0%) patients, ileus in 4 (4/100, 4.0%) patients, respiratory infection in 4 (4/100, 4.0%) patients, deep vein thrombosis (DVT) in 3 (3/100, 3.0%) patients, wound infection in 3 (3/100, 3.0%) patients, renal dysfunction in 2 (2/100, 2.0%) patients, and urinary tract infection in 2 (2/100, 2.0%) patients. Grades III to V SAEs occurred in 4 (4/100, 4.0%) patients, including 2 (2/100, 2.0%) patients who died within 30 days of acute renal failure, 1 (1/100, 1.0%) patient with blood loss, and 1 (1/100, 1.0%) patient with ascending colon leakage. The detailed information is listed in Table 4.

### 4.6. Survival Analysis of Patients with CC Scores of 0-1

A subgroup analysis was conducted. Seventy-nine (79/100, 79.0%) AOC patients achieved CC scores of 0-1, the mPFS was 67.8 (95% CI: 48.3-87.4) months (Figure 2(b)), and the mOS was 95.2 (95% CI: 44.4-146.0) months (Figure 3(e)).

## 5. Discussion

The treatment of AOC remains an open and critical question. Despite clinical remission after palliative surgery and platinum/taxane-based systematic chemotherapy [5, 13, 14], the overall survival of patients with PM is very limited. At present, PM is no longer regarded as a form of systemic and widespread metastasis but a locoregional spread of abdominopelvic malignancies [15]. Accordingly, an integrated treatment strategy of CRS+HIPEC has been developed by pioneering oncologists and has become a standard treatment for malignant mesothelioma of the peritoneum and pseudomyxoma peritoneum and selected patients with colorectal cancer [16–19]. Gradually, the efficacy of CRS+HIPEC has been supported and promoted by various cancer centers for patients with AOC [20–22], but valuable new information and evidence, which could help in the process of selecting patients, formulating HIPEC regimens, normalizing standard surgical procedures, and evaluating safety, are urgently needed.

We aimed to investigate the efficacy and safety of CRS+HIPEC as a first-line surgery strategy in100 patients with AOC. In essence, by comparing long-term survival, we were able to evaluate whether CRS+HIPEC, as a comprehensive therapy strategy, can be a suitable for the routine treatment for AOC. The results showed that the mOS was 87.6 months, and the 1-, 3-, and 5-year survival rates were 94.1%, 68.2%, and 64.2%, respectively. Complete CRS was achieved in 79.0% of patients, and the mPFS was 67.8 months. Of special note are four patients with high-grade serous disease who achieved an OS > 10 years and were disease-free at the time of the most recent follow-up. The multimodality approach following traditional therapy achieved a mOS of 50 months and a mPFS of 3 to 4 months, 5-year survival rates of over 30%, and a relapse rate of 75%. Our results indicated that CRS+HIPEC significantly prolongs the survival of patients with AOC compared to traditional treatment.

van Driel et al. [10] reported the first large-sample randomized controlled trial (RCT) on CRS+HIPEC in primary stage III OC in 2018. A total of 245 patients with newly diagnosed AOC (stage III), fallopian tube carcinoma, and primary peritoneal carcinoma were treated with CRS+HIPEC (DDP 100 mg, 40°C, and 90 min) following neoadjuvant chemotherapy. The mOS was 45.7 months for 106 (87.0%) patients with CC scores of 0-1, and the SAE rate was 27.0%. In our study, a mOS of 87.6 months and a mPFS of 67.8 months were achieved, and these results were better than those in previous studies. Two points deserve special attention. First, our patients underwent radical resection with strict surgical procedures to strive for complete CRS. This leaves a minimal residual tumor burden after surgery. Second, complete and nearly complete CRS was immediately followed by HIPEC with drug combinations at a temperature of 43°C. The synergistic effects of DDP, DTX, hyperthermia, and radical resection could produce significantly better survival benefits not comparable to those of any other treatment modalities applied individually.

The more extensive surgery to minimize tumor burden led to the success of CRS+HIPEC, which is an independent prognostic factor for patients with AOC [23]. In recent years, CRS+HIPEC has also been evaluated in the settings of primary AOC in several studies with variable results, showing that the mOS after complete CRS that achieves a CC score of 0-1 was 32.9 to 79.5 months, and the 5-year survival rate was 12.0 to 66.0% [9, 23–27] (Table 5). Our study also showed favorable survival for patients with CC scores 0-1 who had a mOS of 95.2 months, and the 1-, 3-, and 5-year survival rates were 95.4%, 78.7%, and 73.8%, respectively. Additionally, the multivariate Cox regression analysis identified the CC score as an independent factor for better survival, which is in accordance with the literature reports. In addition, compared with a CC score of 2-3, a CC score 0-1 was approximately 3.2 times more likely to indicate improved survival. All of these results indicated that complete CRS was key to better survival.

As such, every attempt should be made to achieve complete CRS, which means a high risk for AEs and mortality. The safety of CRS+HIPEC has been fully verified, with a perioperative mortality rate of 0 to 10.0% and an incidence of SAEs of 22.0% to 28.0% [18, 34–37]. In our experience, the incidence of grades I to V AEs was 31.0%, and 4.0% of patients developed SAEs, with a perioperative mortality rate of 2.0%, which is similar to that in literature reports. Although we observed no significant differences in patient survival, the mOS of patients without AEs was 53.2 months longer than that of patients with AEs (Figure 3(f)). Hence, further studies are necessary to confirm that AEs may have a detrimental impact on survival.

Apart from its retrospective design, the limitations of this study are the use of single-center cohort with a short median follow-up, but the results showed a tendency towards long survival and safety benefits for AOC patients who underwent CRS+HIPEC as an upfront surgery strategy at experienced high-volume peritoneal cancer centers.

## 6. Conclusions

In summary, this study has provided evidence that CRS+HIPEC, as a preferred surgical strategy, could prolong the survival of AOC patients, especially for those with a KPS score > 80, low PCI, and complete CRS. Therefore, strict patient selection and complete CRS in specialized peritoneal cancer centers are key factors for better survival.

## Figures and Tables

**Table 1:** Demographic and clinical characteristics of 100 AOC patients.

Items	Value
Clinical characteristics	
Age (median, range) (y)	58.5 (28-87)
KPS score (median, range)	80 (50-100)
History of chemotherapy (*n*, %)	
Yes	52 (52.0)
No	48 (48.0)
Histopathology (*n*, %)	
Serous carcinoma	91 (91.0)
Other types	9 (9.0)
Cycles of SC before surgery (median, range)	3 (0-45)
Cycles of IPC before surgery (median, range)	1 (0-9)
Cycles of SC after surgery (median, range)	4 (0-26)
Cycles of IPC after surgery (median, range)	6 (0-8)
SC before surgery (*n*, %)	48 (48)
IPC before surgery (*n*, %)	16 (16)
SC after surgery (*n*, %)	76 (76)
IPC after surgery (*n*, %)	46 (46)
CRS+HIPEC relevant parameters	
Organ regions resected (*n*, %)	
1-3 resections	39 (39.0)
>4 resections	61 (61.0)
Peritoneal regions resected (*n*, %)	
0-3 resections	37 (37.0)
4-6 resections	40 (40.0)
>6 resections	23 (23.0)
Number of anastomosis (*n*, %)	
0-1	34 (34.0)
≥1	66 (66.0)
PCI score (*n*, %)	
≤19	53 (53.0)
>19	47 (47.0)
CC score (*n*, %)	
0-1	79 (79.0)
2-3	21 (21.0)
Lymph node dissection (*n*, %)	
Pelvic lymph node	100 (100)
Abdominal aortic lymph node	68 (68)
Iliac lymph nodes	100 (100)
Fluid output volume at surgery (median, range)	
Blood loss (ml)	550 (0-3,000)
Urine output (ml)	1,500 (300-4,500)
Ascites (ml)	270 (0-8,000)
≤1000 (*n*, %)	68 (68.0)
>1000 (*n*, %)	32 (32.0)
Fluid intake volume at surgery (median, range)	
Plasma (ml)	600 (0-4,000)
RBC (U)^a^	2 (0-12.0)
Other fluids (ml)^b^	6,735 (100-13,950)
CRS+HIPEC duration (median, range) (min)	600 (80-910)
Stay in hospital (median, range) (d)	27 (0-120)

^a^1 U = 200 ml. ^b^Including crystalloid, colloidal fluid injection volume.

**Table 2:** Univariate and multivariate analyses on predictors of OS for 100 AOC patients.

Variables	Univariate analysis				Multivariate analysis			
	*χ* ^2^	*P*	HR	95% CI	*χ* ^2^	*P*	HR	95% CI
CC score (CC 2-3 *vs.* CC 0-1)	11.4	<0.001	4.2	1.8-9.8	6.8	0.009	3.2	1.3-7.5
Age (>58 y *vs.* ≤58 y)	4.8	0.021	2.7	1.1-6.5				
KPS score (≥80 *vs.* <80)	5.0	0.015	0.3	0.1-0.9				
PCI score (>19 *vs.* ≤19)	3.8	0.049	2.5	1.0-6.0				
Ascites (>1000 ml *vs.* ≤1000 ml)	4.2	0.037	2.3	1.0-5.0				

CI: confidence interval; HR: hazard ratio.

**Table 3:** Major clinicopathological features of 4 patients with OS over 10 years.

No	Age (y)	KPS score	Histopathology	CRS	HIPEC	PCI score	CC score	Recurrence	Survival status	OS (mo)
1	41	100	High-grade serous carcinoma	Hysterectomy and resection of bilateral ovarian/fallopian tubes, pelvic lymphadenectomy, ascending colon resection, sigmoidectomy, left and right diaphragmatic peritoneum, greater/lesser omentectomy, round ligament of liver resection, right paracolic sulci peritoneum, mesenteric fulguration	DDP120 mg + DTX 120 mg	19	1	No	Survival	149.2
2	52	90	High-grade serous carcinoma	Hysterectomy and resection of bilateral ovarian/fallopian tube, pelvic lymphadenectomy, greater/lesser omentectomy	MMC 40 mg	13	1	No	Survival	129.9
3	49	80	High-grade serous carcinoma	Hysterectomy and resection of bilateral ovarian/fallopian tube, pelvic lymphadenectomy, sigmoidectomy, cholecystectomy, greater/lesser omentectomy	DDP120 mg + DTX 120 mg	11	1	No	Survival	121.5
4	32	80	High-grade serous carcinoma	Hysterectomy and resection of bilateral ovarian/fallopian tubes, pelvic lymphadenectomy, greater/lesser omentectomy	DDP120 mg + DTX 120 mg	15	1	No	Survival	120.9

MMC: mitomycin C; DDP: cisplatin; DTX: docetaxel; y: year; mo: months.

**Table 4:** Adverse events rate of 100 AOC patients.

Items	*n*, %
SAE (grades III-V)	4 (4.0)
Perioperative mortality	2 (2.0)
Blood loss	1 (1.0)
Colon leakage	1 (1.0)
AE (grades I-II)	27 (27.0)
Anemia and hypoproteinemia	5 (5.0)
Urinary fistula	4 (4.0)
Ileus	4 (4.0)
Respiratory infection	4 (4.0)
DVT	3 (3.0)
Wound infection	3 (3.0)
Renal dysfunction	2 (2.0)
Urinary tract infection	2 (2.0)

AE: adverse event; SAE: serious adverse event; DVT: deep venous thrombosis.

**Table 5:** Previously published studies for AOC patients with complete CRS in recent 5 years.

No.	Author	Year	No. (%)	mOS (mo)	mPFS (mo)	SAE (%)	Mortality (%)
1	Coccolini et al. [31]	2015	54 (100.0)	32.9	12.5	35.2	5.6
2	Kocic et al. [32]	2016	28 (96.8)	51.0	19.0	NA	NA
	Sun et al. [33]	2016	28 (60.9)	79.5	8.5	10.0	0.0
3	Manzanedo et al. [34]	2017	59 (97.0)	NA	17.0	NA	NA
4	Magge et al. [35]	2017	68 (90.6)	41.8	13.3	NA	NA
5	Pavlov et al. [2]	2017	112 (97.0)	40.3	26.7	9.5	0.8
6	Di Giorgio et al. [36]	2017	371 (72.6)	52.4	16.6	17.4	0.0
7	Mendivil et al. [24]	2017	68 (100.0)	33.8	25.1	0.0	0.0
8	Mercier et al. [16]	2018	155 (92.5)	69.3	30.3	NA	NA
9	van Driel et al. [10]	2018	106 (87.0)	45.7	14.2	27.0	0.0
10	This study	2019	79 (79.0)	95.2	67.8	4.0	2.0

NA: not available; mo: months.

## Data Availability

The datasets used and analyzed during the current study are available from the corresponding author on reasonable request.

## Abbreviations

OC: Ovarian cancer
PC: Peritoneal carcinoma
AOC: Advanced ovarian cancer
CRS: Cytoreductive surgery
HIPEC: Hyperthermic intraperitoneal chemotherapy
ULN: Upper limit of normal
ALT: Alanine aminotransferase
AST: Aspartic aminotransferase
Scr: Serum creatinine
KPS: Karnofsky performance status
CA 125: Carbohydrate antigen 125
CA 199: Carbohydrate antigen 199
CEA: Carcinoembryonic antigen
CT: Computed tomography
DTX: Docetaxel
MMC: Mitomycin C
DDP: Cisplatin
SC: Systematic chemotherapy
IPC: Intraperitoneal chemotherapy
OS: Overall survival
PFS: Progress-free survival
AEs: Adverse events
SAEs: Serious adverse events
PCI: Peritoneal cancer index
CC: Completeness of cytoreduction
CIs: Confidence intervals.
