# Corrigendum to “Cytoreductive Surgery plus Hyperthermic Intraperitoneal Chemotherapy Improves Survival with Acceptable Safety for Advanced Ovarian Cancer: A Clinical Study of 100 Patients”

**DOI:** 10.1155/2021/9789613

**Published:** 2021-08-23

**Authors:** Jue Zhang, Xin-bao Li, Zhong-he Ji, Ru Ma, Wen-pei Bai, Yan Li

**Affiliations:** ^1^Department of Peritoneal Cancer Surgery, Beijing Shijitan Hospital, Capital Medical University, China; ^2^Department of Gynecology, Beijing Shijitan Hospital, Capital Medical University, China

In the article titled “Cytoreductive Surgery plus Hyperthermic Intraperitoneal Chemotherapy Improves Survival with Acceptable Safety for Advanced Ovarian Cancer: A Clinical Study of 100 Patients” [1], there was an error in Figure 2(b) where mPTS should have been mPFS and mOS should have been mPFS. The corrected Figure 2 is as below.

## Figures and Tables

**Figure 1:**
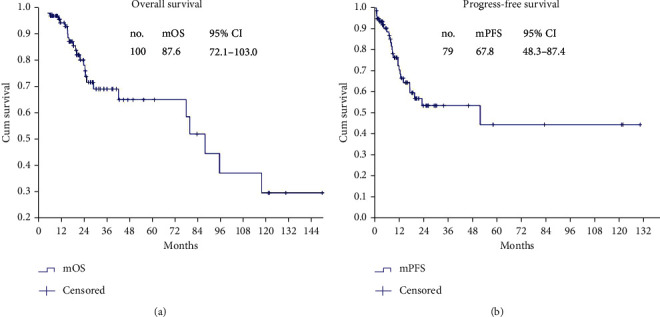
OS and PFS in the AOC patients. (a) OS of 100 AOC patients. (b) PFS of 79 AOC patients with complete CRS+HIPEC.
